# Determination of relationships between placental characteristics and birth weight in Morkaraman sheep

**DOI:** 10.5194/aab-63-39-2020

**Published:** 2020-02-06

**Authors:** Selçuk Özyürek, Doğan Türkyilmaz

**Affiliations:** 1 Department of Veterinary, Erzincan Binali Yıldırım University, Erzincan, Turkey; 2 Department of Animal Science, Faculty of Agriculture, Atatürk University, Erzurum, Turkey

## Abstract

The aim at this study was to determine the relationships among the lamb birth weight, the average cotyledon surface area (ACSA) and cotyledon size. Data were collected from 101 ewes. The general linear model and Pearson correlation coefficient were used for statistical comparison and determination of relationships between variables. Average birth weight (BW), placental weight (PW), cotyledon number (CN), placental efficiency (PE), cotyledon density (CD), cotyledon efficiency (CE) and ACSA were 4.175±.09, 448.8±13.4, 53.34±1.9, 9.65±.3, 0.125±.00, 10.66±.34 and 7.81±.19 cm${}^{2}$, respectively. There was no difference between BW and PE for parity; however, PW, CN, CD, CE and ACSA were affected (p<0.05) by parity. ACSA was found to be the lowest (7.33±.99) with a parity of 2 and the highest (8.61±1.5) with a parity of 4. Birth type affected significantly BW, CN, CD (p<0.05), PW (p<0.001) and ACSA (p<0.01). As the parity progressed, cotyledon depth (CDe) (0.74±.30) and cotyledon width (CWi) (2.64±.46) increased. ACSA, which is a new parameter for uterine capacity, had positive correlations with BW (0.498; p<0.01), PW (0.415; p<0.01), large cotyledon number (CNl) (0.685; p<0.01), cotyledon length (CL) (0.932; p<0.01), CWi (0.920; p<0.01) and cotyledon depth (0.388; p<0.01). The most important finding of this study was the positive correlation between the birth weight and the average cotyledon surface area. This study indicates that average cotyledon surface area and cotyledon size traits (CL and CWi) may be more effective parameters to produce heavier lambs. In conclusion, it is thought that lamb deaths will decrease as a result of triggering placental development with proper feeding during pregnancy. For this purpose, it is recommended to conduct new studies examining the relationship between pregnancy feeding and ACSA. **Highlights.**

- Cotyledon number and cotyledon density decrease with parity, while CE increases.
- The birth type has a significant effect on BW, PW, CN and CD.
- There are positive correlations between the placental weight and large cotyledon number, cotyledon length and cotyledon width.
- ACSA can be used as an important parameter to increase the weight of lambs.
- Especially in multiple birthing, ACSA has been found to be a more determinant index to express uterine capacity instead of PE or CE.

## 1 Introduction

Lambs (litter size, growth performance, etc.) are indicators of the fertility of sheep. Both genetic management and healthy livestock management have a significant role in the survival ability of lambs (Hinch and Brien, 2014).

The population of the fat-tailed Morkaraman breed is approximately 10 million in Turkey. The Morkaraman breed used in this study is known for its insufficient reproduction efficiency, small size, adaptation to marginal conditions, and low wool and milk production. The breeders keep the sheep in the low body condition during the mating season. The breeders do not want twin births in Morkaraman sheep, because twin lambs have lower birth weight (BW), lower survival rate and higher mortality (Ozyurek et al., 2018).

The placenta is a temporary tissue that develops only for the development of the offspring between the chorionic and uterine mucosa of the offspring, and it is formed only during pregnancy. The tasks of the placenta are to feed or nourish and develop the embryo, to provide respiration and discharge, to carry away the metabolic residues and gas, to act as an immunological barrier, and to release hormones during pregnancy (Sen et al., 2013). Owing to the importance of the placenta for the improvement of the offspring, many studies related to the effect of a lot of parameters on this subject have been carried out by researchers (Redmer et al., 2004).

Koyuncu and Duymaz (2017) reported that the highest lamb mortality rate was observed in the postpartum period, especially during the first weeks of the lambing period, that about 20 % of the lambs died before the weaning period, and that 80 % of these deaths happened within the first 10 d of life.

Studies have shown that there is an important correlation among placental characteristics and birth weight in sheep and goat. In previous studies, birth weight was obtained with positive correlations to placental weight for lambs (Echternkamp, 1993), to cotyledon number (CN) for calves (Konyalı et al., 2007), and to placental efficiency (Alkass et al., 2013; Ozyurek, 2019) for kids. Additionally, Kaulfuss et al. (2000) reported that birth type affected the placental weight. Dwyer et al. (2005) also suggested that parity influenced the birth weight and placental traits in sheep. Ocak et al. (2014) noticed that there was a positive correlation between cotyledon weight and cotyledon efficiency in sheep. In the latest studies, instead of determining the cotyledon number and cotyledon weight, the total or average surface area of cotyledons on the placenta was accepted as a new method. On the other hand, other studies have shown that the cotyledon sizes had significant effects on the lamb or kid birth weight (Ocak et al., 2015; Sen and Onder, 2016).

Numerous studies about the placenta generally have shown that there has been a relationship between placental weight, cotyledon efficiency, and birth type and parity. But there is no study on sheep related to the association between parity and birth type, average cotyledon surface area, or cotyledon size. Considering the high correlation between low birth weight and lamb mortality, it is thought that determining the relationship between lamb birth weight and cotyledon surface area or cotyledon size would be a new method of reducing lamb deaths.

**Table 1:** Effect of parity, birth type and sex on placental traits (X±SD).

	N	BW (g)	PW (g)	CN	PE	CD	CE	ACSA (cm${}^{2})$
Parity		n.s.	${}^{*}$	${}^{*}$	n.s.	n.s.	${}^{*}$	${}^{*}$
2	16	3.91 ± .53	397.3 ± 86.7${}^{a}$	57.92 ± 11.8${}^{b}$	10.88 ± 3.1	0.152 ± .04	10.03 ± 1.7${}^{a}$	7.33 ± .99${}^{a}$
3	9	3.82 ± .35	589.1 ± 202.7${}^{b}$	62.17 ± 10.6${}^{b}$	8.87 ± 2.3	0.126 ± .08	10.26 ± .2.0${}^{a}$	7.89 ± 1.6${}^{a}$
4	59	4.06 ± .82	536.6 ± 156.4${}^{b}$	59.65 ± 17.1${}^{b}$	9.95 ± 2.6	0.120 ± .04	10.61 ± .2.9${}^{a}$	8.61 ± 1.5${}^{b}$
5	6	4.10 ± .82	575.8 ± 202.3${}^{b}$	57.83 ± 15.3${}^{b}$	9.35 ± 1.2	0.106 ± .03	11.66 ± .2.8${}^{b}$	8.06 ± 1.2${}^{b}$
6	11	3.54 ± 1.1	434.0 ± 80.2${}^{a}$	44.60 ± 15.4${}^{a}$	8.20 ± 2.7	0.107 ± .04	10.88 ± .4.8${}^{a}$	7.95 ± .98${}^{a}$
Birth type of ewes		${}^{*}$	${}^{***}$	${}^{*}$	n.s.	${}^{*}$	n.s.	${}^{**}$
Single	87	4.106 ± .09	445.7 ± 17.7	53.16 ± 2.1	9.53 ± .3	0.126 ± .40	10.48 ± .40	7.78 ± .19
Twin	14	3.657 ± .22	767.8 ± 40.3	67.04 ± 4.8	9.84 ± .9	0.094 ± .01	11.91 ± .91	9.46 ± .44
Gender of lambs		${}^{***}$	n.s.	n.s.	n.s.	n.s.	${}^{*}$	${}^{*}$
Female	59	3.627 ± .14	587.77 ± 28.1	62.28 ± 3.2	9.32 ± .6	0.116 ± .01	10.51 ± .59	8.38 ± .31
Male	56	4.141 ± .15	576.4 ± 30.0	56.61 ± 3.4	10.17 ± .6	0.110 ± .01	11.74 ± .63	8.66 ± .33
Mean		4.175 ± .09	448.8 ± 13.4	53.34 ± .1.9	9.65 ± .3	0.125 ± .00	10.66 ± .34	7.81 ± .19

Means with different superscript in each column (a, b) differ significantly; n.s. – not significant; ${}^{*}$ P<0.05, ${}^{**}$ P<0.01, ${}^{***}$ P<0.001.

## 2 Materials and methods

The data were collected from 101 Morkaraman sheep in the 2018 lambing season in Erzincan, Turkey (39${}^{\circ }$80${}^{\prime }$ N, 40${}^{\circ }$03${}^{\prime }$ E; 1617 m above sea level). In the study, the placenta, which is a type of animal waste, was studied. Therefore, ethics committee permission was not taken, because the study was conducted on animal wastes. No application was made to cause pain to the animals. During the mating period, sheep were grazed on pasture for 10 h daily. From the 30th day of pregnancy, all sheep were housed in a feedlot environment and fed 500 g of concentrate per sheep per day, as well as dried grass, hay and water ad libitum.

Birth weight and lamb sex were recorded within approximately 12 h after birth. After filtering the placental fluid, placental weight (PW) was measured and recorded. Placental cotyledons were identified and counted, and the total number of cotyledons was recorded as three separate groups according to diameter (CNs, CNm and CNl for numbers of small, medium and large cotyledons, respectively) (Ocak et al., 2015). The cotyledon length (CL), cotyledon width (CWi) and cotyledon depth (CDe) of the 30 cotyledons of the same size chosen from the placenta were measured by digital compass. Placental efficiency (PE), cotyledon density (CD) and cotyledon efficiency (CE) were determined with the formula BW (g)/PW (g), CN/PW (g) and BW (g)/TCSA (total cotyledon surface area), respectively (Konyalı et al., 2007). TCSA was calculated according to the following formula: [((CWi+CL)/4)×2] ×π× TCN, where TCN is total cotyledon number (Sen and Onder, 2016; Ocak et al., 2015). Average cotyledon surface area (ACSA) was obtained by dividing TCSA by cotyledon number.

The effects of ewe parity, birth type and sex on placental and cotyledon traits were analyzed using a completely randomized design by the general linear model (GLM) procedure in SPSS software. Additionally, lamb sex was used as a cofactor in the model (Sen and Onder, 2016). Duncan statistics were computed for the post hoc analysis. To determine the relationships between placental and cotyledon traits, the Pearson correlation test was used. P≤0.05 was assumed as the significant level.

## 3 Results

**Figure 1:**
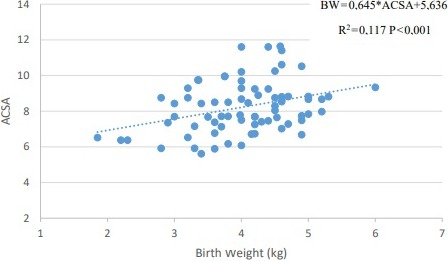
Correlation of lamb birth weight with the ACSA.

Effects of parity, birth type and sex on placental traits with means are shown in Table 1. Average BW, PW, CN, PE, CD, CE and ACSA were 4.175±.09 kg, 448.8±13.4 g, 53.34±0.19, 9.65±0.30, 0.125±0.00, 10.66±0.34 and 7.81±0.19, respectively. There were no differences between parities in BW, CD and PE; however, PW, CN, CE and ACSA were affected by parity (P<0.05). Even if not significant, the lowest birth weight was observed in the group with a parity of 6 (3.54±1.14 kg), and the highest birth weight in the group with a parity of 5 (4.10±.82). ACSA was lowest in the group with a parity of 2 (7.33±.99) and highest with a parity of 4 (8.61±1.5). Also, CN was lowest in the group with a parity of 6. In the regression analysis, a significant relation was found between birth weight and ACSA at the p<0.002 level (y=0.645x+5.636) (Fig. 1).

Birth type affected BW, CN, CD (p<0.05), PW (p<0.001) and ACSA (p<0.01). The lowest BW (3.657±0.22 kg) and CD (0.094±0.01) and the highest PW (767.8±40.3 g), CN (67.04±4.8), PE (9.84±0.9), CE (11.91±0.91) and ACSA (9.46±0.44) were determined in twin births. The sex, on the other hand, had significant effects on BW (p<0.001), CE (p<0.05) and ACSA (p<0.05).

The group with a parity of 3 had the highest CN value (p<0.05); also, even if not statistically significant, this parity had the highest number of CNl (number of large cotyledons) (14.67±2.5) and CNs (number of small cotyledons) (4.50±3.7) (p>0.05) (Table 2). As the parity progressed, the CDe and CWi increased. Although CNs and CNm (number of medium cotyledons) were not affected by birth type, they had significant effects on CNl (p<0.001), CL, CWi and CDe (p<0.01). Single and twin births had 6.7±1.1 CNl and 23.3±2.7 CNl, respectively. Even though there were differences in favor of females for CNs and CNm, they were not significant. The highest CL (3.04±.11) was found in males (p<0.05).

**Table 2:** Effect of parity, birth type and sex on cotyledon traits (X±SD).

	N	CNs	CNm	CNl	CL (mm)	CWi (mm)	CDe (mm)	
Parity		n.s.	n.s.	n.s.	n.s.	${}^{*}$	n.s.	
2	16	3.69 ± 2.1	49.08 ± 14.3	4.62 ± 1.2	2.82 ± .68	2.25 ± .47${}^{a}$	0.54 ± .18	
3	9	4.50 ± 3.7	42.50 ± 21.0	14.67 ± 2.5	3.20 ± .93	2.42 ± .71${}^{a,b}$	0.72 ± .25	
4	59	4.35 ± 9.2	43.95 ± 15.6	10.60 ± 1.7	3.33 ± .76	2.60 ± .59${}^{b}$	0.63 ± .24	
5	6	2.33 ± 1.0	48.17 ± 14.5	7.33 ± 1.8	3.35 ± .92	2.40 ± .42${}^{a,b}$	0.72 ± .33	
6	11	3.20 ± 4.4	32.20 ± 11.3	8.40 ± 1.9	3.20 ± .51	2.64 ± .46${}^{b}$	0.74 ± .30	
Birth type of ewes		n.s.	n.s.	${}^{***}$	${}^{**}$	${}^{**}$	${}^{**}$	
Single	87	3.12 ± 1.1	43.32 ± 2.2	6.7 ± 1.1	2.74 ± .06	2.21 ± .06	0.58 ± .03	
Twin	14	4.28 ± 2.5	39.44 ± 5.1	23.3 ± 2.7	3.31 ± .15	2.71 ± .14	0.85 ± .07	
Gender of lambs		n.s.	n.s.	n.s.	${}^{*}$	n.s.	n.s.	
Female	59	4.95 ± 1.6	41.68 ± 3.5	15.6 ± 1.8	2.94 ± .11	2.39 ± .10	0.70 ± .05	
Male	56	3.26 ± 1.7	38.31 ± 3.8	15.0 ± 2.0	3.04 ± .11	2.47 ± .10	0.68 ± .05	
Mean		3.22 ± .39	43.17 ± 2.22	6.94 ± 0.8	2.76 ± .07	2.21 ± .06	0.59 ± .03	

Means with different superscript in each column (a, b) differ significantly; n.s. – not significant; ${}^{*}$ P<0.05, ${}^{**}$ P<0.01, ${}^{***}$ P<0.001.

In Table 3 Pearson correlation coefficients of placental and cotyledon traits are shown. Also, significant and positive correlations were calculated between BW and CNl (0.323; p<0.01), CL (0.505; p<0.01), CWi (0.414; p<0.01), PE (0.518; p<0.01) and ACSA (0.498; p<0.01). ACSA had positive correlations with BW (0.498; p<0.01), PW (0.415; p<0.01), CNl (0.685; p<0.01), CL (0.932; p<0.01) CWi (0.920; p<0.01) and CDe (0.388; p<0.01). However, there were negative correlations between ACSA and CN (-0.504; p<0.01), CNs (-0.353; p<0.01), CNm (-0.630; p<0.01) and CD (-0.559; p<0.01). The results, as shown in Fig. 1, indicate that a linear relationship was found between birth weight and ACSA in this study (BW = 0.645× ACSA + 5.636; $R^{2}=0.117$, p<0.001).

**Table 3:** Pearson correlation coefficient of placental and cotyledon traits.

Trait	BW	PW	CN	CNs	CNm	CNl	CL	CWi	CDe	PE	CD	CE
PW	.202											
CN	.128	-.127										
CNs	-.166	-.278${}^{*}$	.425${}^{**}$									
CNm	-.006	-.165	.930${}^{**}$	.323${}^{**}$								
CNl	.323${}^{**}$	.299${}^{*}$	-.295${}^{*}$	-.235	-.488${}^{**}$							
CL	.505${}^{**}$	.420${}^{**}$	-.544${}^{**}$	-.378${}^{**}$	-.685${}^{**}$	.749${}^{**}$						
CWi	.414${}^{**}$	.345${}^{*}$	-.383${}^{**}$	-.272${}^{*}$	-.474${}^{**}$	.509${}^{**}$	.715${}^{**}$					
CDe	.130	.125	-.218	.047	-.276${}^{*}$	.213	.342${}^{*}$	.378${}^{**}$				
PE	.518${}^{**}$	-.702${}^{**}$	.183	.096	.130	-.009	-.023	-.025	-.027			
CD	-.037	-.663${}^{**}$	.788${}^{**}$	.584${}^{**}$	.751${}^{**}$	-.389${}^{**}$	-.576${}^{**}$	-.456${}^{**}$	-.201	.556${}^{**}$		
CE	.223	.005	-.756${}^{**}$	-.434${}^{**}$	-.631${}^{**}$	.145	.214	.016	.160	.158	-.512${}^{**}$	
ACSA	.498${}^{**}$	.415${}^{**}$	-.504${}^{**}$	-.353${}^{**}$	-.630${}^{**}$	.685${}^{**}$	.932${}^{**}$	.920${}^{**}$	.388${}^{**}$	-.026	-.559${}^{**}$	.128

${}^{*}$ P<0.05, ${}^{**}$ P<0.01.

## 4 Discussion

This study was conducted to determine the effects of parity, birth type and sex on placental and cotyledon traits in Morkaraman sheep. Parity did not affect BW, and results were similar to findings by Alkass et al. (2013); results were different from Dwyer et al. (2005) and Ocak et al. (2013). Although the explanation of this situation is difficult, we can say that while 60 % of adult live weight for Morkaraman sheep is reached at 14–16 months of age, sheep are only pregnant at ages of 22–24 months (at approximately 90 % of adult live weight), because they are seasonally estrus. Therefore, parity may not have a significant effect on birth weight.

The capacity of the uterus is defined by the total placenta mass that the mother is able to carry. Placental efficiency has been put forward as an index of uterine capacity, especially for higher birth weight, such as for goat, pig and sheep (Konyalı et al., 2007). Previous resources demonstrated that PE increased with parity in sheep (Dwyer et al., 2005; Meirelles, 2017). However, similar to the present study, Konyalı et al. (2007), Ocak et al. (2013), and Sen and Onder (2016) reported that PE did not alter with the parity of doe and ewe. A major cause of the decrease of PE may be explained by the low ratio of PW to BW in ewes with a parity of 3.

The results of this study showed that CN and CD decreased with parity, while CE increased in the same parameter. Similarly, Konyalı et al. (2007), Ocak et al. (2013), and Sen and Onder (2016) reported that there was a reverse relationship between parity and CD. On the other hand, CN was the highest for smaller parities.

Ocak et al. (2014) determined that CE was a reliable criterion for determining the adequacy and capability of the placenta. The cotyledon surface area is a strong sign of the linkage, and it predicts the effectiveness of placental nutrition for the birth of the fetus (Sen and Onder, 2016). In the present study, it was an important finding that only ACSA, a new parameter for placental traits, was affected by parity, birth type and sex. Also, Sen and Onder (2016) reported that ACSA was affected by parity in goats. For parity, the change in ACSA was the same as in PW, too. It has been reported that cotyledon weight is not the main indicator of placental efficiency, and the surface area of the larger cotyledons is a much more efficient parameter than the surface areas of the smaller cotyledons (Ocak et al., 2015). Therefore, it has been reported by the same researchers that ACSA can be used as the main indicator in determining placental efficiency. In our study, while parity had no significant effect on CNs, CNm or CNl, the fact that parity had a significant effect on ACSA supports this result.

In the current study, it was found that the birth type had a significant effect on BW, PW, CN, CD and ACSA. The results related to the effect of birth type were similar to findings by Dwyer et al. (2005) for BW, PW, CN and CD, but they were different from statements by Ocak et al. (2013) for PW and CN. Another important result found in our study is that birth type did not have a significant effect on PE or CE, whereas it had a significant effect on ACSA. This suggests that ACSA is an important sign of uterine capacity in multiple birthing. PW and CN values were higher in twin births compared to single births. Although Ocak et al. (2014) and Sen and Onder (2016) declared that sex was not effective on BW, CE or ACSA in goats, the opposite results were found in the present study.

Similar to this study, Sen and Onder (2016) reported that parity affected CWi, but it did not affect CL in goats. Likewise, Ocak et al. (2013) and Meirelles et al. (2017) stated that parity had an influence on CWi. The maximum number of CNm was observed for a parity of 1. In twins, these were the higher CNl, CL, CWi and CDe, and these results were similar to statements by Ocak et al. (2014) in goat, which are different from findings by Ocak et al. (2013) in ewe. Similar to results by Alkass et al. (2013), Ocak et al. (2014) and Sen and Onder (2016), sex of lamb did not have a significant effect on cotyledon traits except for cotyledon length.

Ocak et al. (2013) and Ocak et al. (2014) reported that there was no significant relation between BW and PW in goat or ewe. In the same way, similar results were observed for correlations between BW and PW. In this study, positive correlations were determined among PW and cotyledon traits (CNl, CL and CWi), but there was a negative correlation between PW and placental traits (PE and CD). Consequently, the findings reported by Ocak and Onder (2011), Ocak et al. (2014), and Sen and Onder (2016) supported the results obtained from the present study. In the properties studied, ACSA had positive correlations (p<0.001) with BW, PW, CNl, CL, CWi, and CDe and negative correlations (p<0.001) with CN and CD. These results were determined for ACSA, which has already been studied in a small number of articles.

## 5 Conclusions

In this study the average cotyledon surface area parameter was used for the first time for ewe placental traits. The considerable findings of the present study were the positive correlation between BW and ACSA. Further, this study indicates that average cotyledon surface area and cotyledon size traits (CL and CWi) may be more effective for producing heavier lambs. Especially in multiple birthing, ACSA has been found to be a more determinant index in order to express uterine capacity instead of PE and CE. Although twin lambs were not wanted by breeders in Morkaraman sheep, the birth type did not affect placental and cotyledon efficiencies. This suggested that the reason for not wanting twin births was because of the low milk production of the ewe rather than low birth weight. In conclusion, it is thought that lamb deaths will decrease as a result of triggering placental development with proper feeding during pregnancy. For this purpose, it is recommended to conduct new studies examining the relationship between pregnancy feeding and ACSA.

## Data Availability

The original data are available upon request to the corresponding author.
